# Sequential changes in serum adipokines and their clinical associations in critically ill COVID-19 patients: A prospective observational study

**DOI:** 10.1097/MD.0000000000050917

**Published:** 2026-10-02

**Authors:** Şahin Temel, Serap Şahin Ergül, Ali Yeşiltepe, Recep Civan Yüksel, Ahmet Safa Kaynar, Murat Sungur, Kürşat Gündoğan

**Affiliations:** a Department of Internal Medicine, Division of Intensive Care Medicine, Erciyes University School of Medicine, Kayseri, Turkey; b Intensive Care Unit, Ministry of Health, Kayseri City Hospital, Kayseri, Turkey.

**Keywords:** adipokines, COVID-19, critical illness, ICU, inflammation, malnutrition, metabolic dysregulation, mNUTRIC

## Abstract

Adipokines regulate metabolic and inflammatory pathways and may be altered during critical illness. We explored sequential serum adipokine concentrations and their associations with nutritional and clinical variables in critically ill COVID-19 patients. This prospective observational, exploratory study included 30 critically ill COVID-19 patients and 10 age- and sex-matched healthy controls. Serum adiponectin, resistin, GLP-1, visfatin, IGF-1, leptin, and acylated ghrelin were measured at intensive care unit admission and on day 7. Biomarker-clinical correlations were analyzed using Spearman correlation, and the Benjamini–Hochberg false discovery rate procedure was applied to account for multiple biomarker comparisons. After false discovery rate correction, longer time to nutrition initiation remained inversely associated with day-7 adiponectin (ρ = −0.464, *q* = 0.027), GLP-1 (ρ = −0.568, *q* = 0.015), IGF-1 (ρ = −0.488, *q* = 0.022), leptin (ρ = −0.539, *q* = 0.015), and acylated ghrelin (ρ = −0.503, *q* = 0.021). Several adipokines showed exploratory associations with nutrition-initiation time in this small cohort of critically ill COVID-19 patients. These observational findings do not establish that earlier nutritional support causes changes in adipokine concentrations and require validation in larger, adequately controlled studies.

## 1. Introduction

Malnutrition significantly affects outcomes in critically ill intensive care unit (ICU) patients, increasing the risk of prolonged mechanical ventilation (MV), infections, extended stays, and mortality. Timely and appropriate nutritional support is essential to reduce these risks and improve outcomes.^[1]^ Following evidence-based guidelines, such as those from the European Society for Clinical Nutrition and Metabolism (ESPEN), is crucial in managing ICU nutrition effectively.^[2]^

Adipokines, including adiponectin, resistin, visfatin, and ghrelin, are bioactive molecules derived from adipose tissues that regulate metabolic and inflammatory processes.^[3]^ Emerging evidence suggests that serum levels may serve as potential biomarkers for assessing the nutritional status and systemic inflammation in critically ill patients.^[4]^ However, the relationship between early nutritional intervention and the dynamics of adipokine levels in patients with acute critical illnesses, particularly those infected with SARS-CoV-2, remains inadequately explored.^[5]^

The modified Nutrition Risk in the Critically Ill (mNUTRIC) score is widely used to evaluate nutritional risk in ICU patients. Elevated mNUTRIC scores have been associated with worse outcomes; however, their relationship with adipokine levels is not well understood. Furthermore, prolonged MV has been implicated in metabolic dysregulation. However, its association with adipokine dynamics requires further investigation.^[6]^

The principal research question was whether serum adipokine concentrations differed between ICU admission and day 7 in critically ill coronavirus disease 2019 (COVID-19) patients and from values observed in healthy controls. Accordingly, sequential changes in adipokine concentrations and comparisons with healthy controls constituted the principal analyses. Associations between adipokine concentrations and nutrition-initiation time, nutritional risk (mNUTRIC score), MV duration, and clinical outcomes were considered exploratory secondary analyses. No formal prespecified hypothesis was defined for these exploratory associations; therefore, these analyses were considered hypothesis-generating.

## 2. Material and methods

### 2.1. Patient and control study group

This prospective study was conducted at the Medical ICU of Kayseri City Hospital from September to November 2022. The study included 30 patients aged 18 years or older, along with a control group comprising 10 healthy volunteers. The age and sex of the control group without comorbidities were matched to those of the patients. No a priori sample-size calculation was performed; the study was therefore considered exploratory and hypothesis-generating. The day-7 eligibility requirement reflected the study design, which required paired biomarker sampling at ICU admission and on day 7. Consequently, patients who died, were discharged, or were transferred before day 7 could not contribute to the serial analysis, which may introduce survivor/selection bias. The healthy control group served as a reference for adipokine concentrations in individuals without COVID-19 or critical illness; without a non-critically ill COVID-19 comparator, differences cannot be attributed specifically to COVID-19 or to critical illness.

### 2.2. Selection of control group

The control group consisted of 10 healthy individuals matched with the patient group in terms of age and sex with no known comorbidities. The healthy controls served as a reference for adipokine concentrations in individuals without COVID-19 or critical illness. However, in the absence of a non-critically ill COVID-19 comparator group, the observed patient-control differences cannot be attributed specifically to COVID-19 or to critical illness. Therefore, the healthy control group should be considered a reference group rather than a disease-specific comparator.

### 2.3. Sample collection and laboratory analyses

The study population comprised patients with laboratory-confirmed SARS-CoV-2 infection who had been followed up in the ICUs with respiratory failure for more than 24 hours. Diagnosis was confirmed by polymerase chain reaction assay of nasal and pharyngeal swabs or endotracheal aspirate, in accordance with the World Health Organization guidelines.^[7]^ Blood samples were obtained from patients at ICU admission and on the 7th day. Blood samples were collected concurrently with routine blood samples obtained from the patients at 10:00. The samples were centrifuged at 5000 rpm for 10 minutes within a 30-minute timeframe. Laboratory analyses of human Acylated ghrelin (AG), adiponectin, glucagon-like peptide-1 (GLP-1), insulin-like growth factor-1 (IGF-1), leptin, resistin, and visfatin proteins were performed using enzyme-linked immunosorbent assay (ELISA) kits in accordance with the procedures of the Erciyes University Biochemistry Department Laboratory.

**ELISA measurement of adipokines:** ELISA KIT, Bioassay Technology Laboratory, Shanghai, China.

**Acylated ghrelin:** Catalogue code: E3090Hu, Human Acylated ghrelin; **Adiponectin:** Catalogue code: E1550Hu Human Adiponectin; **Ghrelin:** Catalogue code: E3091Hu Human Ghrelin; **GLP-1:** Catalogue code: E0022Hu Human GLP-1; **IGF-1:** Catalogue code: E0103Hu Human IGF-1; **Leptin:** Catalogue code: E1559Hu Human Leptin; **Resistin:** Catalogue code: E0338Hu Human Resistin; **Visfatin:** Catalogue code: E0025Hu Human Visfatin.

Data on age, sex, height, weight, body mass index, Acute Physiology and Chronic Health Evaluation score, and modified Nutrition Risk Score (mNUTRIC) were collected. Patients were monitored for 7 days following admission to the ICU. Glasgow Coma Scores were recorded after admission to the ICU, and Sequential Organ Failure Assessment scores were calculated daily. MV status and vasopressor requirements were documented. Daily dietary patterns and calorie intakes were recorded. The length of stay in the ICU and hospital, duration of MV, and data on mortality in the ICU and hospital were recorded. The timing, route, dose, and composition of nutrition were adjusted according to the guidelines published by the European Society for Clinical Nutrition and Metabolism (ESPEN) Association in 2023.^[2]^

### 2.4. Statistical analysis

IBM Statistical Package for the Social Sciences (SPSS) 13.0 (IBM Corp., Armonk) and GraphPad Prism were used for data analysis. Continuous variables with a normal distribution were expressed as mean ± standard deviation, whereas non-normally distributed continuous variables were expressed as median (25th–75th percentile, interquartile range [IQR]). The Shapiro–Wilk test was used to assess normality. Pearson correlation analysis was used for normally distributed continuous variables, and Spearman rank correlation analysis was used for non-normally distributed continuous variables. Baseline-to-day-7 adipokine changes within patients were assessed using the Wilcoxon signed-rank test. Patient-versus-healthy-control comparisons were performed using the Mann–Whitney *U* test. Friedman testing was used to evaluate repeated daily nutritional measurements during the 7-day follow-up period. No a priori sample-size calculation was performed. Therefore, given the limited sample size, the study was considered exploratory and hypothesis-generating. The sequential changes in adipokine concentrations from ICU admission to day 7 and the comparisons with healthy controls constituted the principal analyses. Associations between adipokine concentrations and nutritional or clinical variables were considered exploratory secondary analyses. To account for multiple biomarker comparisons, the Benjamini–Hochberg false discovery rate (FDR) correction was applied separately within each clinically defined family of exploratory biomarker comparisons. Both nominal *P* values and FDR-adjusted *q* values were reported, and *q* < 0.05 was considered statistically significant after correction for multiple testing. Correlation coefficients were reported as measures of effect size, with approximate 95% confidence intervals for the principal Spearman correlations. Given the limited sample size (n = 30), multivariable models including several potential confounders were considered prone to overfitting and were therefore not performed. Baseline and day-7 adipokine measurements were available for all 30 ICU patients. Missing data were not imputed, and analyses were performed using available cases. Accordingly, the analysis involving total MV duration included 29 patients.

### 2.5. Ethical standards

All procedures performed in studies involving human participants or on human tissue were in accordance with the ethical standards of the institutional and/or national research committee and with the 1975 Helsinki Declaration and its later amendments or comparable ethical standards. The study was approved by the Non-Interventional Clinical Research Ethics Committee of Erciyes University School of Medicine on May 05,2021, with protocol number 2021/357. Prior to enrollment, patients or their first-degree relatives were provided with an explanation of the study and were asked to sign a written consent form. Individuals whose consciousness improved during their stay in the ICU were re-consented to participate. Patients who declined to participate at any stage were excluded from the study.

## 3. Results

This study included 30 critically ill patients and 10 healthy controls. All 30 patients included in the analytic cohort completed the planned day-7 biomarker sampling and had complete baseline and day-7 measurements for the 7 principal adipokines. No biomarker data were imputed. The mean age of the patients was 70 ± 16 years, and 43% were female. The median APACHE-II scores of patients were 20.5 (IQR = 15.75–28), and the mean mNUTRIC score was 5 ± 1. The need for vasopressors and invasive mechanical respiratory support (MV) was 30% and 40%, respectively. The median duration of MV was 3 days (IQR: 2–14) (Table 1).

**Table 1:** Demographic and clinical information of the patients.

	Patient, n = 30	Healthy control, n = 10
Age, mean ± SD, yr	70 ± 16	68 ± 11
Gender, n (%) Female	13 (43)	4 (40)
BMI, median (IQR), kg/m^2^	25 (22–31)	26 (23–32)
mNUTRIC score, mean ± SD	5 ± 1	
APACHE II, median (IQR)	20.5 (15.75–28)	
SOFA score, median (IQR)	6 (5–8)	
Vasopressor, n (%)	9 (30)	
Mechanical ventilation (MV), n (%)	12 (40)	
Duration of MV, median (IQR), d	3 (2–14)	
Initiation time of feeding, median (IQR), h	2 (1,75–3)	
Length of stay in ICU, median (IQR), d	10 (7–18)	
Length of hospital stay, median (IQR), d	14 (10–21)	
ICU mortality, n (%)	15 (50)	
Hospital mortality, n (%)	16 (53)	

APACHE II = Acute Physiology and Chronic Health Evaluation II, BMI = body mass index, ICU = intensive care unit, IQR = interquartile range, mNUTRIC = modified Nutrition Risk in the Critically Ill, MV = mechanical ventilation, SD = standard deviation, SOFA = Sequential Organ Failure Assessment.

Table 2 shows the median calorie intake of the patients. On the day of admission, 4 patients did not receive any nutrition, while 26 patients received some route of nutritional therapy. The patients with the highest median calorie intake were patients 1 (1332, min–max:1296–1818), 26 (1440 min–max:580–1584), and 30 (1452 min–max:0–1652; Table 2).

**Table 2:** The daily calorie intake of patients.

Patients number	Daily calorie intake, kcal/d							Median calorie intake, (min–max)
	Day 1	Day 2	Day 3	Day 4	Day 5	Day 6	Day 7	
1	1332	1442	1818	1296	1296	1728	1296	1332 (1296–1818)
2		180	300	300	300	400	400	300 (0–400)
3	480	1008	960	864	1152	336	864	864 (336–1152)
4	1680	576	768	300	600	800	900	768 (300–1680)
5	150	200	100	1800	1260	756	800	756 (100–1800)
6	400	400	400	500	600	400	400	400 (400–600)
7	600	800	1000	1000	600	600	650	650 (600–1000)
8	180	432	540	504	600	450	600	504 (180–600)
9	1008	1152	934	1156	1152	444	600	1008 (444–1152)
10	500	600	500	800	1000	800	900	800 (500–1000)
11	1152	1152	1152	1152	1152	1152	1152	1152 (1152–1152)
12	468	612	1080	972	1620	1134	1100	1080 (468–1620)
13	672	1380	1552	1552	400	400	400	672 (400–1552)
14	400	600	500	600	500	500	500	500 (400–600)
15	300	400	500	400	450	500	500	450 (300–500)
16	816	1416	684	864	840	900	800	864 (684–1416)
17	720	1728	1296	1152	1152	1152	1152	1152 (720–1728)
18	192	576	1152	1152	984	912	900	912 (192–1152)
19		100	576	576	800	576	576	576 (0–800)
20	864	864	864	864	864	864	468	864 (468–864)
21	384	1152	1152	1152	1668	600	1000	1152 (384–1668)
22			300	600	600	650	700	600 (0–700)
23	540	600	700	900	700	800	800	700 (540–900)
24		300	250	200	200	300	400	250 (0–400)
25	900	1000	600	700	800	800	800	800 (600–1000)
26	580	1452	1302	1360	1440	1440	1584	1440 (580–1584)
27	640	876	876	548	700	1000	1000	876 (548–1000)
28	816	1152	1152	1404	924	870	1050	1050 (816–1404)
29	400	500	400	500	600	300	400	400 (300–600)
30		1168	528	1452	1652	1552	1500	1452 (0–1652)

Upon admission, 13% of patients received enteral tube feeding. By the 7th day of follow-up, this figure had increased to 27%. In terms of parenteral nutrition, 27% of patients received parenteral nutrition upon admission, but this rate decreased to 10% by the 7th day of follow-up. Patients who were conscious and did not need MV were fed a hospital diet, which accounted for 60% to 63% of all cases (Table 3).

**Table 3:** Daily nutrition type of patients.

Patients	Enteral tube feeding, n (%)	Parenteral nutrition, n (%)	Oral supplement/diet, n (%)
Baseline	4 (13)	8 (27)	18 (60)
Day 2	5 (17)	7 (23)	18 (60)
Day 3	5 (17)	6 (20)	19 (63)
Day 4	6 (20)	5 (17)	19 (63)
Day 5	7 (24)	4 (13)	19 (63)
Day 6	7 (24)	4 (13)	19 (63)
Day 7	8 (27)	3 (10)	19 (63)

PN = parenteral nutrition.

The median daily calorie intake per kilogram of weight of patients during their ICU stay was 6.6 to 11 kcal/kg/d. On day 5 of the 7-day follow-up in the ICU, the patients reached a maximum calorie intake of 11 kcal/kg/d.

Compared with healthy controls, serum adiponectin levels were significantly lower in patients both at ICU admission (*P* = .0087, *q* = 0.021) and on day 7 (*P* = .0017, *q* = 0.0079). Serum resistin levels were also significantly lower at admission (*P* = .0091, *q* = 0.021) and on day 7 (*P* < .0001, *q* = 0.00056). Day-7 GLP-1 (*P* = .0063, *q* = 0.021) and visfatin (*P* = .00024, *q* = 0.0017) levels were significantly lower in patients after FDR correction. Although baseline GLP-1, baseline visfatin, day-7 IGF-1, and baseline acylated ghrelin showed nominally significant differences in the unadjusted analyses, these differences did not remain statistically significant after FDR correction. The serum adipokine levels of the patients and healthy controls are shown in Figure 1.

**Figure 1.**
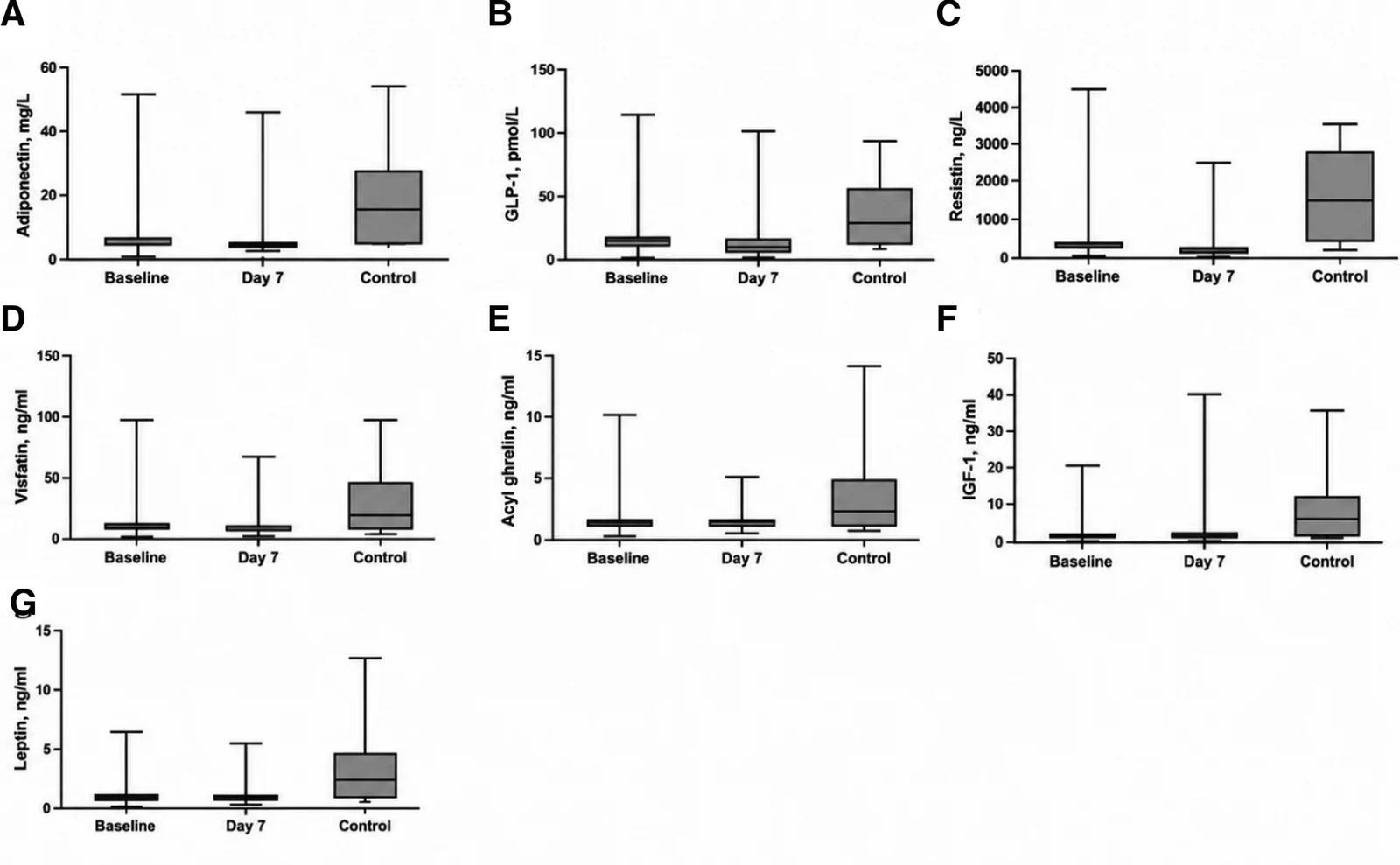
Serum adipokine concentrations in critically ill COVID-19 patients and healthy controls. Serum adipokine concentrations in critically ill COVID-19 patients at ICU admission and on day 7 compared with healthy controls. After Benjamini–Hochberg false discovery rate (FDR) correction, adiponectin levels were significantly lower in patients at ICU admission (*P* = .0087, *q* = 0.021) and on day 7 (*P* = .0017, *q* = 0.0079). Resistin levels were also significantly lower at ICU admission (*P* = .0091, *q* = 0.021) and on day 7 (*P* < .0001, *q* = 0.00056). Day-7 GLP-1 (*P* = .0063, *q* = 0.021) and visfatin (*P* = .00024, *q* = 0.0017) levels were significantly lower in patients than in healthy controls. Differences in baseline GLP-1, baseline visfatin, day-7 IGF-1, and baseline acylated ghrelin were nominally significant in the unadjusted analyses but did not remain statistically significant after FDR correction. (A) Adiponectin, (B) GLP-1, (C) resistin, (D) visfatin, (E) acylated ghrelin, (F) IGF-1, and (G) leptin. COVID-19 = coronavirus disease 2019, FDR = false discovery rate, GLP-1 = glucagon-like peptide-1, ICU = intensive care unit, IGF-1 = insulin-like growth factor-1.

Exploratory correlation analysis showed inverse associations between time to nutrition initiation and several day-7 adipokines. After Benjamini–Hochberg FDR correction across the 14 baseline and day-7 biomarker comparisons, significant inverse associations remained for day-7 adiponectin (ρ = −0.464, 95% CI: −0.706 to −0.125, *P* = .0097, *q* = 0.027), GLP-1 (ρ = −0.568, 95% CI: −0.771 to −0.262, *P* = .0010, *q* = 0.0147), IGF-1 (ρ = −0.488, 95% CI: −0.722 to −0.156, *P* = .0062, *q* = 0.0216), leptin (ρ = −0.539, 95% CI: −0.753 to −0.222, *P* = .0021, *q* = 0.0149), and acylated ghrelin (ρ = −0.503, 95% CI: −0.731 to −0.175, *P* = .0046, *q* = 0.0214). The association between baseline leptin and time to nutrition initiation was nominally significant (ρ = −0.368, *P* = .0455) but did not remain significant after FDR correction (*q* = 0.106; Fig. 2).

**Figure 2.**
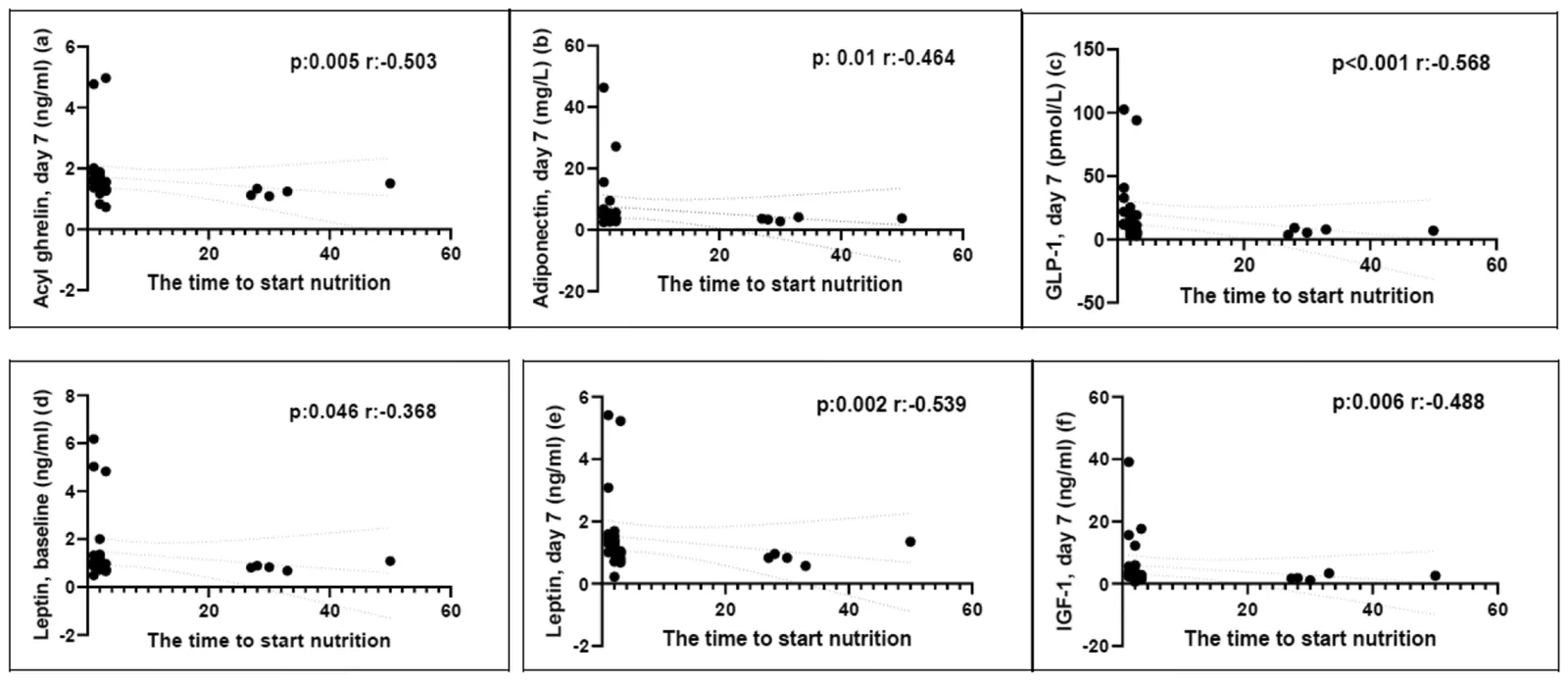
Correlation between time to nutrition initiation and adipokine levels on day 7 in ICU COVID-19 patients. Correlations between time to nutrition initiation and day-7 serum adipokine concentrations in critically ill COVID-19 patients. Spearman’s ρ coefficients indicate the direction and strength of the associations. The corresponding nominal *P* values and false discovery rate-adjusted *q* values are reported in the main text. COVID-19 = coronavirus disease 2019, GLP-1 = glucagon-like peptide-1, ICU = intensive care unit, IGF-1 = insulin-like growth factor-1.

Day-7 acylated ghrelin was positively correlated with total MV duration (n = 29, ρ = 0.497, 95% CI: 0.160 to 0.731, *P* = .0061); however, this association did not remain statistically significant after FDR correction (*q* = 0.085). The relationships between nutrition-initiation time and serum adipokine levels are shown in Figure 2, and the association between day-7 acylated ghrelin and total MV duration is shown in Figure 3.

**Figure 3.**
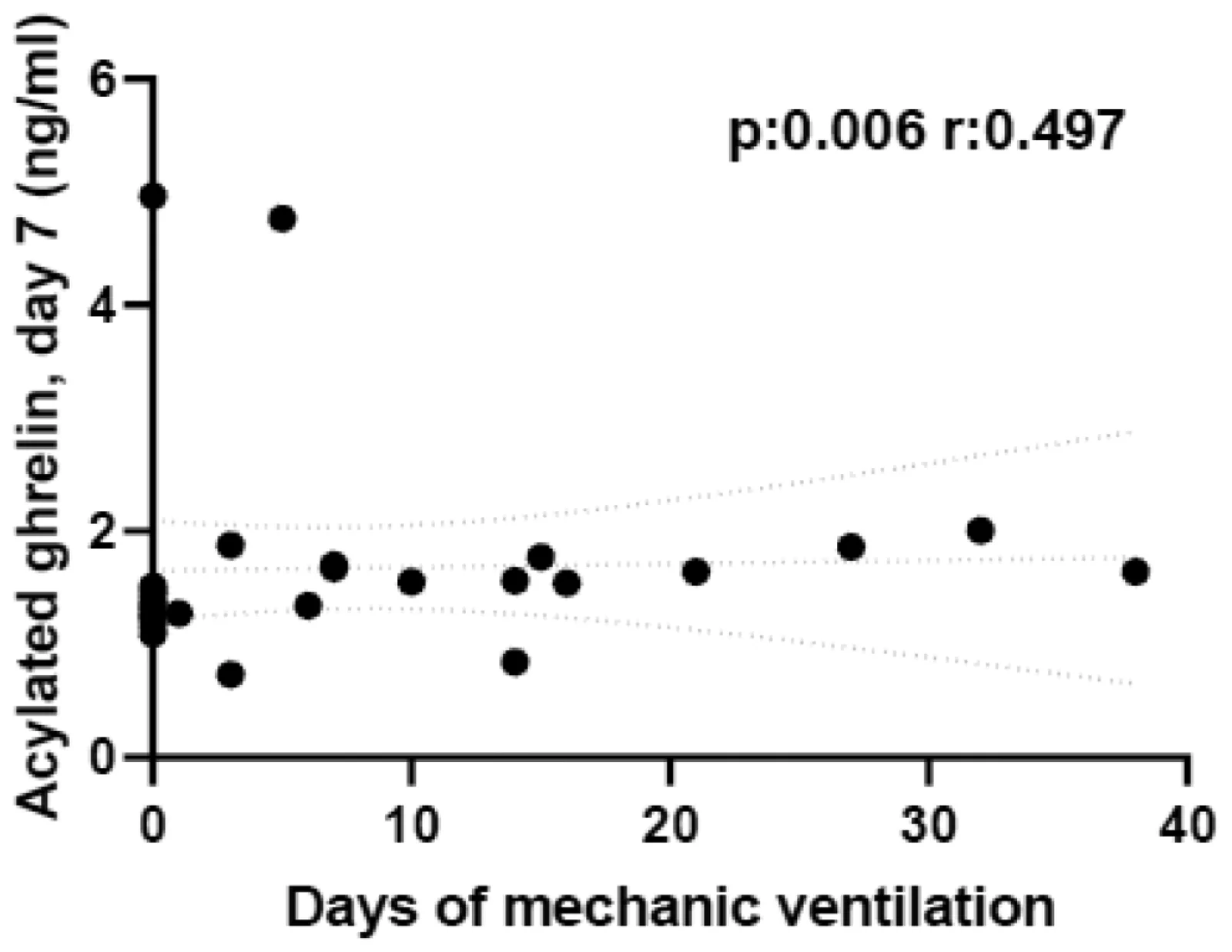
This figure shows the association between serum acylated ghrelin (AG) levels on day 7 and total mechanical ventilation (MV) duration in critically ill COVID-19 patients (n = 29, ρ = 0.497, *P* = .0061). Although this association was nominally significant, it did not remain statistically significant after Benjamini–Hochberg FDR correction (q = 0.085) and should therefore be interpreted as exploratory. AG = acylated ghrelin, COVID-19 = coronavirus disease 2019, FDR = false discovery rate, MV = mechanical ventilation.

The median lengths of stay in the ICU and hospital were 10 (IQR: 7–18) and 14 (IQR:10–21), respectively. The ICU and hospital mortality rates were 50% and 53%, respectively (Table 1).

## 4. Discussion

Our study is one of the few to examine sequential changes in adipokine concentrations in critically ill COVID-19 patients and their associations with nutritional and clinical variables. Several adipokines differed between critically ill patients and healthy controls, suggesting alterations in metabolic and inflammatory pathways during critical illness. In the exploratory analyses, longer time to nutrition initiation was inversely associated with day-7 adiponectin, GLP-1, IGF-1, leptin, and acylated ghrelin concentrations after correction for multiple comparisons. These associations should be interpreted cautiously and do not demonstrate a causal effect of nutritional timing on adipokine concentrations.

The anti-inflammatory properties of adiponectin are well documented, yet its levels were significantly reduced in our cohort compared to healthy controls at both baseline and day 7.^[8]^ A study by Flikweert et al found lower plasma adiponectin levels in critically ill COVID-19 patients than in healthy controls, which is consistent with our findings. Unlike our study, they used a single plasma measurement and included patients with lower Acute Physiology and Chronic Health Evaluation II scores (16–21), suggesting less severe illness.^[9]^

GLP-1, an anti-inflammatory hormone, was lower in critically ill patients than in controls; however, after FDR correction, the difference remained statistically significant only on day 7.^[10]^

Resistin promotes inflammation by increasing the levels of pro-inflammatory cytokines such as IL-6 and TNF-α.^[11,12]^ Previous studies have reported higher resistin levels in critically ill patients, correlating with IL-6 levels and disease severity in conditions such as sepsis and ARDS.^[12-14]^ Similarly, increased visfatin and resistin levels have been linked to greater ICU disease severity.^[15]^ However, in our study, baseline and day 7 levels of resistin and visfatin were lower than those in controls, potentially reflecting differences in patient characteristics or treatments.

Leptin, secreted by adipose tissue, regulates weight via the hypothalamus and is elevated in ICU COVID-19 patients.^[9,16,17]^ However, no link to inflammatory conditions, such as endotoxemia or sepsis, has been established.^[18]^

Differences in resistin, visfatin, and leptin concentrations between our cohort and previous studies may reflect differences in patient characteristics, disease severity, or treatment exposures. However, systematic patient-level data on corticosteroid and IL-6 inhibitor use were not available in our dataset; therefore, the potential contribution of these treatments cannot be determined.

In critically ill patients, increased cytokines resulting from trauma, sepsis, or inflammation may lead to low serum AG levels.^[19]^ Our study found that the baseline AG levels were lower than those in the control group. However, there are conflicting results regarding AG levels in critically ill patients.^[20,21]^ This may be due to the fact that the forms of ghrelin analyzed in the studies, such as total and AG, differ.

A 2015 study of 543 critically ill non-COVID-19 patients found that low plasma IGF-1 levels early in ICU admission were linked to ARDS and mortality.^[22]^ In our study, day-7 serum IGF-1 levels were lower in COVID-19 patients than in controls, although this difference did not remain statistically significant after FDR correction.

We observed inverse associations between time to nutrition initiation and several day-7 adipokine concentrations, including adiponectin, GLP-1, IGF-1, leptin, and acylated ghrelin, which remained significant after FDR correction. To our knowledge, this relationship has not been specifically evaluated in critically ill patients. However, the timing of nutritional support was not randomized and may have been influenced by disease severity, gastrointestinal tolerance, clinical decision-making, and other measured or unmeasured factors. Therefore, these findings should be considered exploratory and do not demonstrate that earlier nutritional support caused changes in adipokine concentrations.^[23]^

A rat study showed that ghrelin reduced ventilator-associated lung injury (VILI) by decreasing the expression of TLR4, NF-κB, and pro-inflammatory cytokines.^[24]^ In critically ill patients, a 2010 study found higher serum ghrelin levels in spontaneously breathing patients than in those on MV.^[20]^ In our cohort, the association between day-7 acylated ghrelin and MV duration was nominally significant but did not remain significant after FDR correction. Therefore, this finding should be interpreted as exploratory and requires confirmation in larger cohorts.^[21]^

Our study has several strengths. First, we analyzed 7 serum adipokines at ICU admission and on day 7. Second, evidence has shown that heparin affects plasma adipokine levels^[25-27]^; therefore, serum adipokine levels were analyzed instead of plasma. Additionally, the study explored relatively understudied relationships between serum adipokine concentrations, nutrition-initiation time, and MV duration in critically ill COVID-19 patients.

### 4.1. Limitations

This study has several limitations, including the small sample size and single-center design, which may limit the generalizability of the findings. The lack of subgroup analyses based on COVID-19 phenotypes or comorbidities may have obscured variations in adipokine responses. Systematic patient-level data on corticosteroid exposure, IL-6 inhibitor use, and other major COVID-19 treatments were not available in the study dataset. These unmeasured treatment exposures, together with heterogeneity in nutritional practices, may have influenced inflammatory and metabolic biomarkers and represent potential sources of residual confounding. Given the small sample size and multiple biomarker comparisons, the present study should be considered exploratory and hypothesis-generating. The reported associations do not establish causal effects of nutritional timing or other clinical exposures. Moreover, multivariable adjustment for potential confounders was not performed because of the limited sample size. The requirement for patients to remain available for day-7 sampling may have introduced survivor/selection bias, as patients who died, were discharged, or were transferred before day 7 were not represented in the serial analysis. This may further limit the generalizability of the findings to the overall critically ill COVID-19 population. Furthermore, the absence of a non-critically ill COVID-19 comparator group prevents the separation of COVID-19-specific effects from those related to critical illness. Finally, a complete contemporaneous screening log was not retained during the pandemic recruitment period; therefore, the exact number of patients assessed for eligibility but not enrolled and the individual reasons for non-enrollment could not be reliably reconstructed.

## 5. Conclusion

In this small prospective observational cohort, sequential changes in serum adipokine concentrations were evaluated in critically ill COVID-19 patients. Several adipokines differed between critically ill patients and healthy controls, and day-7 concentrations of adiponectin, GLP-1, IGF-1, leptin, and acylated ghrelin were inversely associated with time to nutrition initiation after correction for multiple comparisons. These findings should be considered exploratory and hypothesis-generating and do not establish that earlier nutritional support causes changes in adipokine concentrations or improves clinical outcomes. Larger, adequately powered prospective studies with appropriate comparator groups and adjustment for relevant confounders are needed to validate these associations and clarify their clinical significance.

## Author contributions

**Conceptualization:** Şahin Temel, Serap Şahin Ergül, Ali Yeşiltepe.

**Data curation:** Şahin Temel.

**Formal analysis:** Şahin Temel, Serap Şahin Ergül.

**Investigation:** Şahin Temel, Serap Şahin Ergül, Recep Civan Yüksel, Ahmet Safa Kaynar.

**Methodology:** Şahin Temel, Serap Şahin Ergül, Murat Sungur, Kürşat Gündoğan.

**Writing – original draft:** Şahin Temel, Serap Şahin Ergül, Ali Yeşiltepe, Recep Civan Yüksel, Ahmet Safa Kaynar, Murat Sungur, Kürşat Gündoğan.

**Writing – review & editing:** Şahin Temel, Serap Şahin Ergül, Ali Yeşiltepe, Recep Civan Yüksel, Ahmet Safa Kaynar, Murat Sungur, Kürşat Gündoğan.
